# A pathogenic variant in the uncharacterized *RNF212B* gene results in severe aneuploidy male infertility and repeated IVF failure

**DOI:** 10.1016/j.xhgg.2023.100189

**Published:** 2023-03-31

**Authors:** Moran Gershoni, Tslil Braun, Ron Hauser, Shimi Barda, Ofer Lehavi, Mira Malcov, Tsvia Frumkin, Yael Kalma, Shmuel Pietrokovski, Eli Arama, Sandra E. Kleiman

**Affiliations:** 1 ARO-The Volcani Center Institute of Animal Science, Bet Dagan, Israel; 2 Racine IVF Unit and Male Fertility Clinic and Sperm Bank, Lis Maternity Hospital, Tel Aviv Sourasky Medical Center, affiliated with the Sackler Faculty of Medicine, Tel Aviv University, Tel Aviv, Israel; 3 Department of Molecular Genetics, Weizmann Institute of Science, Rehovot, Israel

**Keywords:** Aneuploidy, IVF failures, Human infertility, RNF212B, Meiosis, Genome instability

## Abstract

Quantitative and qualitative spermatogenic impairments are major causes of men’s infertility. Although *in vitro* fertilization (IVF) is effective, some couples persistently fail to conceive. To identify causal variants in patients with severe male infertility factor and repeated IVF failures, we sequenced the exome of two consanguineous family members who underwent several failed IVF cycles and were diagnosed with low sperm count and motility. We identified a rare homozygous nonsense mutation in a previously uncharacterized gene, *RNF212B*, as the causative variant. Recurrence was identified in another unrelated, infertile patient who also faced repeated failed IVF treatments. scRNA-seq demonstrated meiosis-specific expression of *RNF212B*. Sequence analysis located a protein domain known to be associated with aneuploidy, which can explain multiple IVF failures. Accordingly, FISH analysis revealed a high aneuploidy rate in the patients' sperm cells and their IVF embryos. Finally, inactivation of the *Drosophila* orthologs significantly reduced male fertility. Given that members of the evolutionary conserved *RNF212* gene family are involved in meiotic recombination and crossover maturation, our findings indicate a critical role of *RNF212B* in meiosis, genome stability, and in human fertility. Since recombination is completely absent in *Drosophila* males, our findings may indicate an additional unrelated role for the *RNF212*-like paralogs in spermatogenesis.

## Teaser

We report the discovery of a pathogenic variant in the uncharacterized human gene *RNF212B*, which belongs to an evolutionary conserved gene family involved in meiotic recombination. RNF212B loss of function is associated with sperm abnormalities and broad aneuploidy in patient-derived sperm cells and embryos, resulting in multiple repeated IVF failures.

## Introduction

Approximately 15% of human couples fail to conceive following a year of attempts.^1^ In about half of the cases, a male factor may account for the infertility.^1^^,^^2^ Whereas it is clear that many of the male infertility cases contain a heritable component,^3^^,^^4^ the genetic etiology for the majority of the cases remains incomplete. Male infertility is usually the consequence of quantitative and/or qualitative spermatogenic impairments. The most frequent quantitative condition is known as oligozoospermia (Oligo), where mature sperm cells are few in number, exhibiting no apparent defects in their morphology and motility. However, in clinical practice, quantitative and qualitative spermatogenesis impairments tend to appear concurrently in infertile men, a condition known as oligoasthenotheratozoospermia (OAT).^5^ As of November 2022, the OMIM database has 82 entries for spermatogenic failure (SPGF), out of which 67 reported autosomal recessive, nine reported autosomal dominant, and six reported X- or Y-linked inheritance. For only 22 of the 82 SPGF genes, Oligo or OAT were reported as a possible outcome. Nevertheless, a systematic literature screening for genes involved in human male infertility and linked to Oligo or OAT, revealed only a small number of known genes.^6^ Consistently, fewer than 20 genes associated with OAT were reported in a recent study associating human genes with various pathozoospermia (refer to the whole spectra of pathological conditions of low semen quality).^7^

In some cases, specific pathozoospermia were associated with recurrent gene mutations; for example, qualitative spermatogenic impairments manifested by specific abnormal sperm morphologies, such as macrozoospermia (sperm cells with abnormally large and misshapen heads), globozoospermia (round-headed sperm cells due to lack of acrosome), multiple morphological abnormalities of the sperm flagella (MMAF), and primary ciliary dyskinesia.^8^ Although medically assisted reproduction (MAR), i.e., *in vitro* fertilization (IVF) and intracytoplasmic sperm injection (ICSI), are great solutions for some men with Oligo or OAT, other couples undergoing MAR treatments repeatedly fail to conceive even after dozens of IVF or ICSI cycles. Furthermore, evaluation of reproductive potential based only on standard semen quality analysis is often inadequate to predict MAR treatment outcomes. Therefore, identifying causal genetic factors in infertile men with Oligo or OAT may accelerate diagnosis, prognosis, and save frustrating repeated futile MAR treatments. Moreover, it can also help evaluate the likelihood of passing the same or *de novo* (when the germline variant affects genome stability) genetic aberrations to the next generation.

Here, we report the discovery of a rare, recessive, nonsense mutation in a previously uncharacterized gene called *Ring Finger Protein 212B* (*RNF212B*), as the probable causative variant of male infertility in two brothers with OAT and repeated unexplained MAR failures. Furthermore, we also identified an identical variant in yet another unrelated individual with recurrent MAR failures. Fluorescence *in situ* hybridization (FISH) analyses of sperm cells and ICSI-produced preimplanted embryos derived from the affected brothers, both revealed a high rate of aneuploidy. Single-cell RNA sequencing (scRNA-seq) data analysis detected relatively high expression of *RNF212B* during meiosis. Inactivation of the three orthologous genes in *Drosophila* resulted in various degrees of male and female infertility, and examination of the affected testes revealed moderate defects in spermatid terminal differentiation. Since during meiosis of spermatocytes in *Drosophila*, homologous chromosomes undergo pairing without crossover and recombination, these findings imply that members of this gene family might also function in the chromosomal pairing process itself or in yet another process during spermatogenesis.^9^^,^^10^^,^^11^

## Methods

### Patients and study population

The present study includes five members of a consanguineous Jewish family of Turkish origin, three of whom underwent exome sequencing. In addition, 338 infertile men, of whom 59 were Sephardic Jewish from a similar origin as the family analyzed (Jewish origin from Turkey, Bulgaria, Greece, or Spain), and 281 from other origins (Israeli Jewish, Arabs, and Christians) underwent mutational screening. A control group of 185 men of similar ethnicities with proven fertility was also included. All patients were referred to the Male Fertility Clinic and Sperm Bank at the Tel Aviv Sourasky Medical Center during 1997–2017and were part of the 1,500 recruited patients who constitute our research patient cohort.

All infertile men underwent Y chromosome microdeletion assessment, and for 19% of them, the karyotype was reported. All men included in the study were queried about their parental origin, family ancestry, and fertility history. Ethnicity was categorized as Arab or Jewish according to the parents' origin. The former belonged to the Muslim, Christian, and Druze communities. The Jews were subdivided according to their country of ancestral origin.

### Ethical approval

All study participants consented to undergo genetic evaluations and signed written informed consent. The local institutional review board committee approved the study in accordance with the Helsinki Declaration of 1975.

### Genetic analysis

Genomic DNA was extracted from white blood cells as detailed.^12^^,^^13^ Exome sequencing was carried out at Theragene using the Illumina Hiseq platform, and the SureSelectXT Human All Exon V6 kit was used for library preparation (target size was 58 Mb). The coverage of ≥ X20 was >95% for the target genome in all samples.

### Bioinformatics analysis

Bioinformatics and genomics analyses were performed as we previously described in detail.^13^^,^^14^^,^^15^ Briefly, adapter sequences and low-quality tails of reads were removed with the software Trimmomatic.^16^ Raw reads were then aligned to the reference genome (hg19) with BWA-MEM.^17^ Then, using Picard Tools software, we removed duplicates, indexed, and compressed the aligned sequence to the BAM format (http://broadinstitute.github.io/picard/). Further sequence data processing was performed using GATK.^18^ The resulting g.vcf files were joint-called with an additional 25 fertile males taken from our exome sequencing database, all of whom underwent sequencing on the same platform and capturing kit, as a negative control (i.e., causative genotypes are not expected to be presented by the fertile control). All detected variants were comprehensively annotated using the Annovar tool kit,^19^ and the variant categories, transcript consequences, functional consequences, population frequencies, evolutionary conservation, and pathology information were added to each variant. Candidate variants were considered if they passed the filtering procedures: Likely causative variants were identified by filtering the data according to (1) the recessive or X-linked mode of inheritance, (2) the expected population frequency of the variant (MAF <1%), and (3) the functional impact of the variant (conservation, loss of function, functional prediction). Finally, the relevance of the phenotype was assessed using comprehensive expression data (as described below), Gene Ontology terms, and model organism data.^20^^,^^21^

To further assess the association between the genes and the pathology of defects in spermatogenesis and sperm maturation, we included gene expression data: RNA-seq transcript per million (TPM) values were retrieved from the GTEx portal.^22^ A testis specificity value was given to each gene as previously described.^13^^,^^23^ Briefly, we create a synthetic vector for exclusive expression in testis. The Pearson product-moment correlation coefficient (r) was calculated for a testis exclusive expression synthetic vector against all the gene expression vectors. Results are displayed as a list of r-values, indicating the testis specificity level, where the closer the r-values to r = 1, the higher the testis specificity. We further obtained bioinformatics-supportive evidence by searching for associations between the candidates' genes and the terms: “azoospermia,” “meiosis,” or “spermatogenesis” using the GeneCards suite that also includes the MalaCards databases.^24^^,^^25^

RNF212-like protein sequences were identified in the NCBI sequence databases by BLAST searches, starting with the two human RNF212 isoforms, using an e-value threshold of 1e-13, and then searching iteratively with the most taxonomically and sequence-similar distant hits. Representative sequences from diverse taxa (Table S5) were used to sample the diversity of RNF212-like proteins. Multiple sequence alignments were done using the GLAM2,^26^ COMPASS,^27^ and LAMA^28^ programs. Dendrograms were calculated using the phyml v 20120412 program with its smart model selection mode.^29^

### Verification of pathogenic variants

Variants suspected to cause OAT were confirmed by Sanger sequencing and by examining the segregation within the family. The primers and conditions for the PCR amplification are provided in Table S1. Variants frequencies were assessed in 338 men selected from our initial cohort of 1,300 infertile men^12^ and 181 fertile men by restriction fragment-length polymorphism (RFLP) analysis for candidate variants. Restriction sites for distinguishing between normal and mutant alleles were identified with the help of the “Webcutter” program (http://www.firstmarket.com/cutter/cut2.html) or manually whenever a mismatch insertion was necessary. Testicular expression of candidate genes was confirmed by RT-PCR of two testicular samples obtained from two men with obstructive azoospermia and normal spermatogenesis (data not shown).

### scRNA-seq bioinformatics analysis

The merged human expression matrix data file from Drop-seq experiments on human testicular cells from four adult males was obtained from Gene Expression Omnibus (GEO: GSE142585) from Shami et al.^30^ Overall, 13,597 cells were used for the analysis. We used Shami et al.^30^ annotation files according to the cell samples' barcodes to identify human testicular cell types. To verify the exact testis cell type the candidate genes are expressed in, we used the expression of the following known RNA markers:

spermatogonia (*GFRA1*, *HORMAD1*, *ID4*, *ITGA6*, *LY6K*, *STRA8*, *SYCP2*, *UCHL1*, and *UTF1*); spermatocytes (*PIWIL1* and *SYCP3*), spermatids (*ACRV1*, *PRM1*, *TNP1*, and *TSSK6*); Leydig cells (*IFG1/2* and *STAR*), endothelial cells (*NOSTRIN* and *VWF*); testicular macrophages (*CD52*, *CD163*, *LYZ*, and *TYROBP*); pericytes (*ADIRF*, *MCAM*, *PDGFRB*, and *STEAP4*); or myoid cells (*ACTA2* and *MYH11*), as done in Hardy et al.^31^ Raw counts were counts per million (CPM) normalized using the edgeR package.^32^ The normalized expression matrix of the marker genes was hierarchically clustered by Pearson correlation coefficient, using the MORPHEUS software (https://software.broadinstitute.org/Morpheus) (Figure S1). Additional scRNA-seq data were obtained from Hermann et al.^33^ to determine the specific stages of *RNF212B* expression during spermatogenesis. The data was uploaded on the 10X Genomics Loupe cell browser (https://support.10xgenomics.com/single-cell-gene-expression), and the median normalized average of all significantly expressed genes in the identified clusters was retrieved. *RNF212B* and its paralog expression in the clusters were determined, and the clusters were annotated according to Hermann et al.^33^

### Fluorescence *in situ* hybridization

In brief, sperm cells were washed with PBS pH 7.4 twice and suspended in freshly prepared cold fixative solution (methanol/acetic acid, 3/1), dropped onto slides (Super Frost/plus; Menzel-Glaser, Braunschweig, Germany), and air-dried. Due to the severe low concentration of sperm cells, sperm cells were localized in the drop before proceeding with the hybridization to ease their detection afterward. Washed slides were incubated for 10 min with a freshly prepared solution of 1M Tris pH 9.5 with 0.25 mM DTT for sperm cell DNA denaturation and washed with SSC, dehydrated, and fixed with fresh 1% formaldehyde. For FISH chromosome pairing detection, the slides (one from the patient and one from a normozoospermic man) were treated according to the manufacturer’s instructions. Triple-color FISH was performed for the detection of chromosomes 18, X, and Y (Cytocell, Cambridge, UK). Only sperm cells with observable tails were counted.

### Microsatellite analysis

Twelve microsatellites were analyzed by ABI PRISM GeneScan using fluorescent primers labeled with FAM for their amplification. Eight markers were upstream from the *RNF212B* gene, up to 2Mb, and four markers were downstream, down to 0.7Mb (provided in Table S2).

### Fly strains

All fly strains were grown at 25°C. *yw* flies were used as wild-type controls. *bam-Gal4* (this driver line was used in a combination of two copies of the driver, one on the X and one on the third chromosome, together with one copy of *UAS-dicer* recombined on the third chromosome) was generated by crossing fly lines obtained from L. Gilboa (WIS, Israel) and from M. Wolfner (Cornell University, USA). *nos-Gal4-VP16* (stock #4937) was obtained from the Bloomington *Drosophila* Stock Center. *narya*^*JJ6*^, *narya*^*G4*^, and *vilya*^*826*^ mutants, as well as the *nenya*^*RNAi*^ line were obtained from R.L. Hawley (Stowers Institute for Medical Research, USA).

### Fertility assays

Mutant adult males (0–1 day old) were mated with *yw* virgin females (3 days old) as singles, while mutant virgin females (3 days old) were mated with *yw* adult males (0–1 day old) as singles. After 3 days together, the parents were removed from the vial, the larval progeny were allowed to develop into adult flies, and the number of adult progeny in each vial was determined. Ten vials were scored for each genotype.

### Immunofluorescence staining of fly testes and the used antibodies

Testes were dissected from young adult flies (0–2 days old) and moved immediately into ice-cold fix solution (4% paraformaldehyde [PFA] diluted in PBS) within a glass well plate positioned on ice. Next, the glass well plate was moved from the ice to a rocker in room temperature (RT) for additional 20 min with slow rotation, rinsed three times for 10 min with PBX (PBS with 0.1% Triton X-100), blocked with PBS/BSA (1% BSA in PBS) for 45–60 min at RT, and incubated with a primary antibody (diluted in PBS/BSA) overnight at 4°C. Testes were then rinsed in PBX, incubated with a secondary antibody for 1 h at RT, rinsed again, and mounted in Vectashield mounting medium with DAPI (Vector Laboratories).

The primary antibodies used in this study are rabbit polyclonal anti-cleaved-*Drosophila* Dcp1 antibody (Asp 216, Cell Signaling Technology; 1:50) and mouse monoclonal anti-polyglycylated tubulin antibody (clone AXO 49, Sigma, 1:5,000). All secondary antibodies were used in a dilution of 1:250 (Jackson ImmunoReaserch). Phalloidin-TRITC (Sigma, 1:500) was used to label F-actin.

## Results

### Clinical assessment of infertile men from a consanguineous Jewish Turkish family

**Figure 1:**
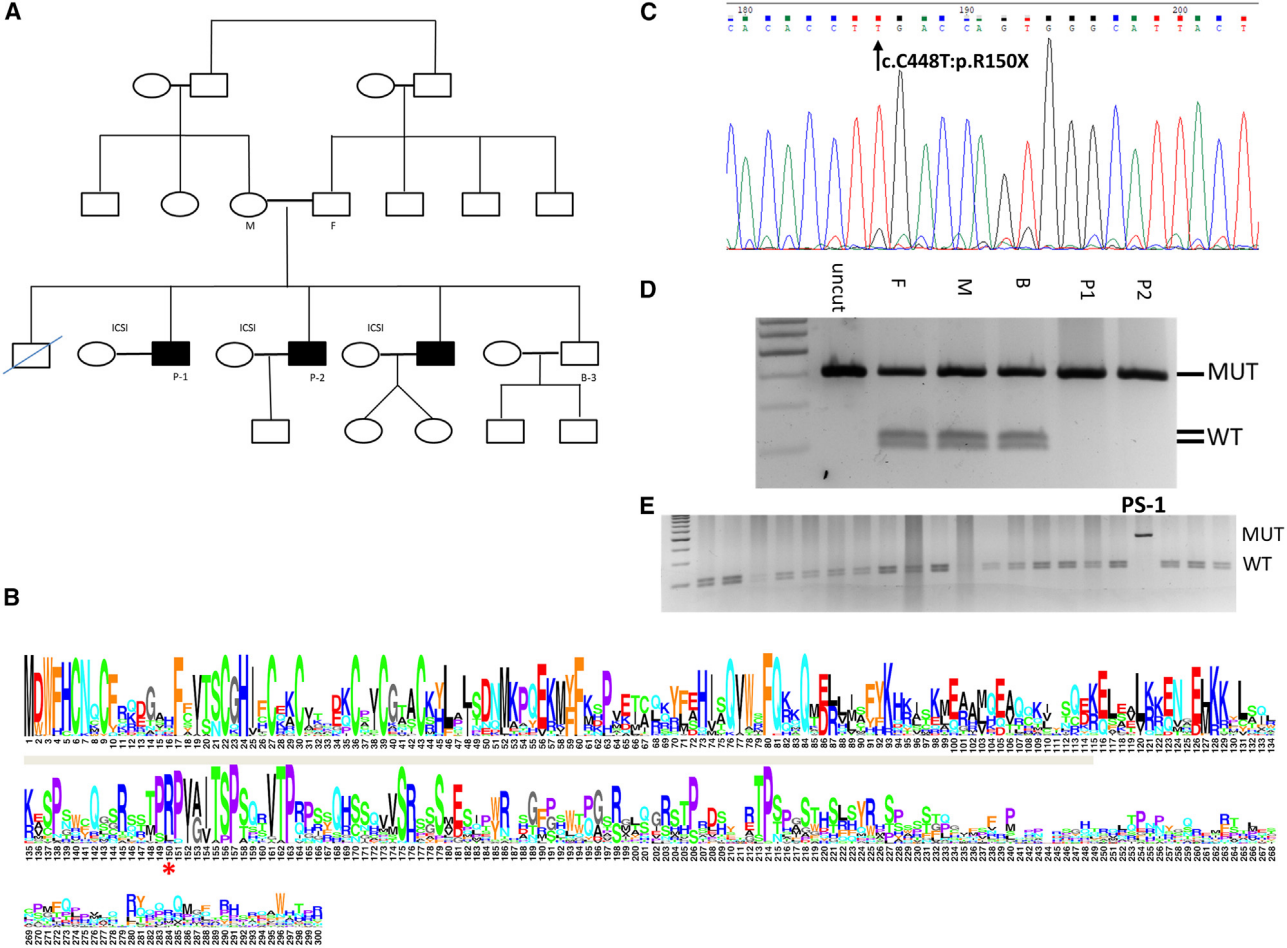
Pedigree, variant validation, and sequence conservation of RNF212B PV R150X (A) Family pedigree. Black fill denoting azoospermic siblings. Affected brothers, P-1 and P-2, and their fertile brother, B-3, underwent whole-exome sequencing. DNA was sampled from the patient’s mother (M) and father (F) for RFLP analysis. (B) Sequence logo of RNF212B proteins (Table S5). Positions are numbered by the human RNF212B protein, with only positions in it shown. The R150X nonsense mutation is marked by a red '**∗**', and the RING zinc-finger domain is marked by a gray bar. (C) Validation of the identified *RNF212B* PV by Sanger sequencing. (D) RFLP screening was performed on the family members (F, father; M, mother; B-3, unaffected brother), which confirmed that the parents are both carriers of the PV. (E) RFLP screening in infertile men identified an additional patient, PS-1 (black asterisk), homozygote to the PV.

To identify causative variants associated with OAT, we used next-generation sequencing on samples from two patient brothers and a third fertile brother, all of whom are consanguineous Jewish Turkish descendants (Figure 1A). Patient 1 (P-1) attended our male fertility clinic and sperm bank (the Tel Aviv Medical Center) for counseling at the age of 33. His clinical record included catheterization and ligation of varicocele, splenectomy after a traffic accident, medically treated high blood pressure, and type 2 diabetes mellitus (T2DM). Both his hormone and karyotype profiles were normal, displaying a Y chromosome without AZF-microdeletion (Table 1). He has been previously diagnosed with OAT, a diagnosis that was reconfirmed in our clinic, and (the couple) underwent 24 unsuccessful IVF cycles, three of which were performed in our center (two cycles with fresh sperm and one with frozen sperm). Although the fertilization success rate with ICSI was 68% (21 of 31 injected oocytes), which is similar to the 70%–85% general success rate, all the attempts to conceive after embryo transfers were unsuccessful. Finally, the couple conceived by sperm donation, which ended a decade of fertility treatments. Of note, at the age of 42, P-1 was diagnosed with chronic renal failure, presumably due to unbalanced T2DM.

**Table 1:** Ethnic and clinical features of the infertile brother analyzed

Pat^a^	Age∗	Ethnic origin	Hormones (mIU/mL)		Testis (mL)		Sperm analysis				Clas^f^	Kar^g^	Clinical findings
			LH	FSH	L	R	Con^b^	Mot^c^	Vit^d^	Mor^e^			
P-1	33	Turkish Jew	4.3	3.5	nr	nr	12	28 bc	80	3	OTA	46XY	catheterization and ligation of varicocele, splenectomy after a traffic accident and high pressure
P-2	30	Turkish Jew	4.8	5.6	4	4	0.19–0.01	30-23 abc	64–52	3	sOTA	46XY	light varicocele at the right testis

Reference values: Follicle-stimulating hormone (FSH 1.3–16 mIU/mL), luteinizing hormone (LH 1.2–10 mIU/mL).

L, left; nr, not reported; R, right.

a Patient.

b Concentration ∗10^6^.

c Motility (%).

d Vitality (%).

e Normal morphology (%).

f Classification (semen).

g Karyotype.

∗ Age at the time of fertility treatments.

The second infertile brother, herein referred to as patient 2 (P-2), attended our clinic at the age of 30, 5 years after P-1 first attended our center, following unreported number of unsuccessful IVF cycles in another center. Physical and ultrasound examinations revealed normal testis volume and light varicocele on the right testicle (stage I-II). Both hormone and karyotype profiles were normal, with no AZF-microdeletion on the Y chromosome. As compared with P-1, P-2 had much lower sperm cell concentration, a typical characteristic of severe OAT (sOAT) (Table 1). The couple underwent eight ICSI cycles in our center with a fertilization success rate of 70% (23 of 33 injected oocytes), and with sufficient or moderate embryo quality. However, all the attempts to conceive following the transfers of 15 embryos in total were unsuccessful. It is noteworthy that when P-2 first attended our clinic, he reported having two older brothers with fertility impairments; these included P-1 and another brother who refused to participate in the study.

### Identifying the causative pathogenic variant in P-1 and P-2

To identify the causative pathogenic variant (PV) in P-1 and P-2, we conducted whole-exome sequencing for the affected siblings and for one of their fertile brothers (B-3). Since the brothers are consanguineous, we reasoned two possible modes of inheritance: homozygosity for a recessive variants or X-linkage inheritance. Several restricting criteria were applied to narrow down potential chromosomal sites of the causative variant: (1) rarity of the variant in available public databases; (2) homozygosity of both of the affected brothers to the variant; (3) absence or heterozygosity of the variant in the fertile brother, as well as in a control group of 25 fertile men (detailed in the methods section); and (4) the variant likely affect the function of the gene product. Following these initial assessments, 15 variants located in 14 genes were considered for follow-up (Table S3). Using the Genotype-Tissue Expression (GTEx) dataset^22^ to reveal tissue-specific RNA expressions of the corresponding genes, two out of the 15 variants were considered the most likely causative PVs based on high expression levels of the corresponding genes in the testis (Table S3).

The first variant is a missense mutation in a gene called *Excision repair cross-complementing 4* (*ERCC4*; OMIM: 133520; GeneBank: NM_005236:exon6:c.A1062C:p.K354N). The variant could also be detected by RFLP analysis in the affected brothers using a mismatched primer as described (Table S1). However, we considered it an unlikely causative variant for infertility for several reasons. The *ERCC4* RNA is expressed to different extents in essentially all 53 different tissues listed in the GTEx dataset, including in the testis, and recent scRNA-seq data^30^ indicates that its peak of expression during spermatogenesis occurs only after meiosis in the round and elongating spermatids (Figure S2). Furthermore, whereas mutations in the *ERRC4* gene have not been reported to be associated with human male infertility, they were associated with Xeroderma Pigmentosum, Fanconi Anemia Complementation Group F (FANCF; OMIM: 603467), and Group Q (FANCQ; OMIM: 615272),^34^^,^^35^^,^^36^ all of which are disorders that have not been associated with our patient brothers. Moreover, the variant associated with these disorders was mapped to *ERCC4* GeneBank: NM_005236 exon 6, which is absent in the two testis-specific *ERRC4* transcript isoforms listed in the GTEx dataset (Figure S2).

The second variant is a nonsense mutation in a previously uncharacterized gene called *Ring Finger Protein 212B* (*RNF212B*; GeneBank: NM_001282322:exon8:c.C448T; Table S3). This variant is a single-base substitution in the *RNF212B* gene, which changes the cytosine in position 448 to thymine (C448T) and accordingly converts the codon for arginine-150 to a premature opal stop codon (R150X), leading to a predicted truncation of the protein C-terminal half. Protein sequence conservation analysis in jawed vertebrates indicates that this arginine residue is highly conserved and positioned within a larger conserved region of the protein (Figure 1B). Validation of the *RNF212B*^*C448T*^ variant was subsequently performed by Sanger sequencing of samples from P-1 and P-2 (Figure 1C). The heterozygosity status of the parents, as well as of the B-3 fertile brother, was confirmed by RFLP analysis (Figure 1D).

To examine whether this variant might be associated with other patients with severe infertility, we turned to our cohort of 1,300 infertile men (and 335 fertile men as control). From the initial cohort, we first obtained all men from the same ethnic group as P-1 and P-2 (i.e., 57 patients and 21 fertile men, all of the Turkish, Greek, and Bulgarian Jewish descent). In addition, we obtained patients from other ethnic groups (255 and 26 of Jewish and non-Jewish descent, respectively, mainly Jews of Ashkenazi descent), who presented a range of quantitative semen impairment conditions ranging from a low number of sperm cells in the ejaculate, similar to P-1 and P-2 (e.g. OAT, Oligo, and sOAT) to extremely low concentration or no sperm cells at all in the ejaculate (e.g., cryptozoospermia and azoospermia [AZO], respectively). We also specifically included 13 infertile men with a history of IVF failure and seven men who reported repeated miscarriages of different Jewish ethnicities. For control, an additional 164 fertile men from matching ethnicities were also included in the screen, as summarized in Table 2. Altogether, we screened 338 patients and 185 fertile men, mostly of Jewish Ashkenazy and Balkans descents, for the presence of the *RNF212B*^*C448T*^ variant using the RFLP assay. Significantly, we identified the same *RNF212B*^*C448T*^ homozygous variant in a patient with OAT, who has been reported to undergo multiple IVF failures (Figure 1E). This patient, termed herein PS-1, is unrelated to P-1 and P-2, as he belongs to a non-reported consanguineous Ashkenazi family (descendant of Czechoslovak-Polish Jews). Accordingly, copy number variants (CNVs) analysis of microsatellites flanking the *RNF212B*^*C448T*^ variant in PS-1, revealed a different genetic background than that of P-1 and P-2 (Table S2). Furthermore, although his karyotype was normal, PS-1 was also found to carry the most frequent partial microdeletion (gr/gr) in the AZFc region, usually manifested by variable semen conditions ranging from normal semen analysis (normozoospermia) to AZO. Finally, PS-1 RFLP analysis for the presence of the *ERCC4* K354N variant was negative.

**Table 2:** Results of the RFLP screen for RNF212B:exon8:c.C448T:p.R150X

Group (size)	Ethnic origin	Total men tested	Semen finding		Men positive for the PV	Mutant alleles
			OAT^b^	AZO		
Fertile^a^	TBGS	21	0	0	0	0
	Other Jews	164	0	0	0	0
	non-Jews	0	0	0	0	0
	Total	185	0	0	0	0
Infertile	TBGS	57	22	35	0	0
	Other Jews	255	176	79	1	2
	non-Jews	26	23	3	0	0
	Total	338	221	117	1	2

TBGS, men from the similar/related ethnicity as the family. According to the history of emigration, Jews of Turkey, Bulgaria, Greece, and Spain originated from Spain.

The 20 men who reported repeated IVF failure (n = 13) or miscarriage (n = 7) are included in the screening according to their ethnic origin.

a Eighty-five fertile patients were normozoospermic and the others were fertile men with at least two children who were not tested by semen analysis.

b OAT includes oligoasthenotheratozoospermic, severe oligoasthenotheratozoospermic, and cryptozoospermic men.

Since the *RNF212B*^*C448T*^ variant appeared in two unrelated Jewish descendants, we assessed its overall population frequency. The initial evaluation indicated an extremely low frequency of heterozygosity to the variant (MAF ≤ 0.0001) in all the accessible public databases (Table S3), with no documented case of homozygosity. However, dividing the Genome Aggregation Database (gnomAD; a database that aggregates whole-exome and genome sequencing data from a variety of sequencing projects^37^) into its subpopulations uncovered an elevated frequency of the variant (as of Jan 2023, MAF 0.0029–0.0037) in Ashkenazi Jews. Nonetheless, it is noteworthy that this variant was not found in our in-house whole-exome database of ∼500 individuals. This database is an aggregate of whole-exome data from various projects. None of the projects has recruited infertile men, and none of the sequenced individuals reported infertility. Therefore, the frequency of infertile individuals is assumed to reflect the incidence of infertility in the population. According to the distribution of reported ethnicity, about 35% of the individuals in the database are Jews of European descent from highly related populations as our patients, e.g., Ashkenazi Jews (∼25%) and Jews descended from the Balkans and Turkey (∼10%).^12^ Therefore, although the *RNF212B*^*C448T*^ variant is considered extremely rare in all the gnomAD subpopulations, it is rare but notably more frequent in Ashkenazi Jews.

### *RNF212B* is expressed during meiosis and affects sperm ploidy

**Figure 2:**
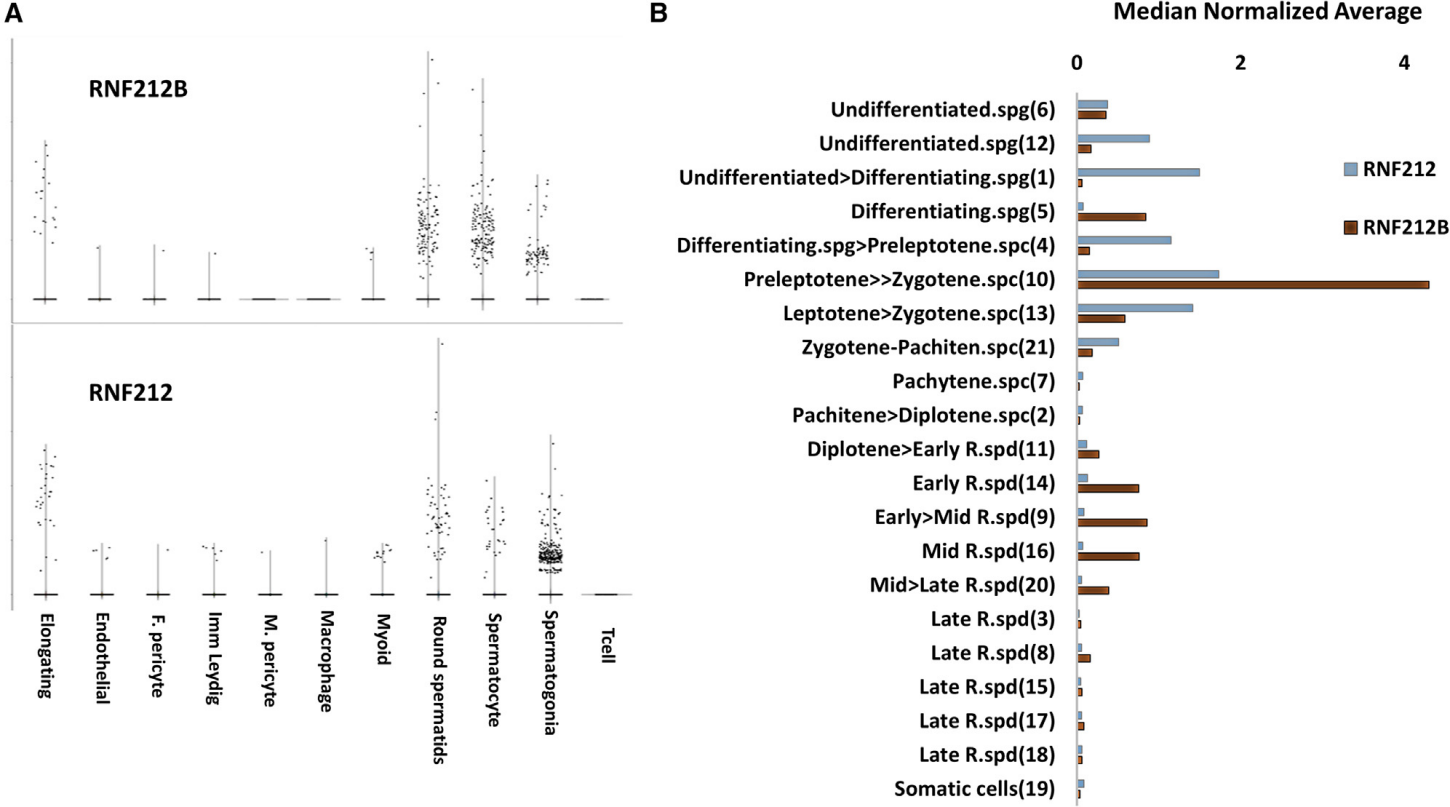
*RNF212B* scRNA-seq analysis (A) Counts per million (CPM) normalized expression levels (y axis) of *RNF212* and *RNF212B* from >13,000 testis cells obtained from Shami et al.^30^ The annotated cell types are denoted on the x axis. (B) The median normalized average expression (x axis) of *RNF212B* (brown bars), and *RNF212* (gray bars). The annotated cell clusters are presented on the y axis, sorted according to their chronological stage during spermatogenesis, as denoted in Hermann et al.^33^ spg, spermatogonia; spc, spermatocyte; R.spd, round spermatids. The affiliated cells cluster number is in parentheses, as annotated by Hermann et al.^33^*RNF212B* is specifically expressed in affiliated meiotic clusters (meiosis prophase I and meiosis II, y axis).

To begin exploring possible molecular mechanisms underlying infertility in men carrying the *RNF212B*^*C448T*^ variant, we first examined tissue expressions of both *RNF212B* and its paralog *RNF212*. Whereas in human tissues, *RNF212B* RNA is almost exclusively expressed in the testis, *RNF212* (OMIM: 612041) expression is more general, displaying relatively elevated levels in the testis and the ovary but also in several somatic tissues (Figure S3). Furthermore, analysis of public human scRNA-seq data^30^ revealed that although expression of both genes is elevated in spermatogonia, the *RNF212B* RNA persists in spermatocytes and round spermatids (late pre-meiotic cells and early post-meiotic cells, respectively), while *RNF212* expression is comparably reduced in these cells (Figure 2A). In the later stages of elongating spermatids, both genes still displayed low expression levels, implying a possible role for these genes in post-meiotic spermatid differentiation (Figure 2A). Similar results were also obtained by analyzing the expression of both genes in another, more refined, scRNA-seq dataset of human spermatogenic cells.^33^ The expression levels of *RNF212B* and *RNF212* are relatively high throughout the leptotene, zygotene, and pachytene stages of meiotic prophase I. The peak of expression of both genes occurs in the pre-leptotene to the zygotene stages, in which the *RNF212B* expression level (median normalized average = 4.3, Loupe p value = 2.7∗10^−21^) is notably higher than that of *RNF212* (median normalized average = 1.72, Loupe p value = 5.9∗10^−8^; Figure 2B). However, initiation of *RNF212* expression is in early pre-meiotic stages during the transition of the undifferentiated spermatogonia cells into the differentiating stages (median normalized average = 1.49, Loupe p value = 5.4∗10^−15^, Figure 2B). In addition, *RNF212B* has a notable second expression phase during late meiosis II, peaking in early to mid-round spermatid (median normalized average = 0.85, Loupe p value = 0.00001, Figure 2B), and is only reduced after meiosis II in the late haploid round spermatids (Figure 2B). In contrast, *RNF212* has no significant expression throughout meiosis II or in round spermatids (Figure 2B). Collectively, the expression patterns of *RNF212B* and *RNF212* support overlapping roles during meiosis prophase I, and non-overlapping roles of these genes during the pre- and the late meiotic stages of human male germ cells.

**Figure 3:**
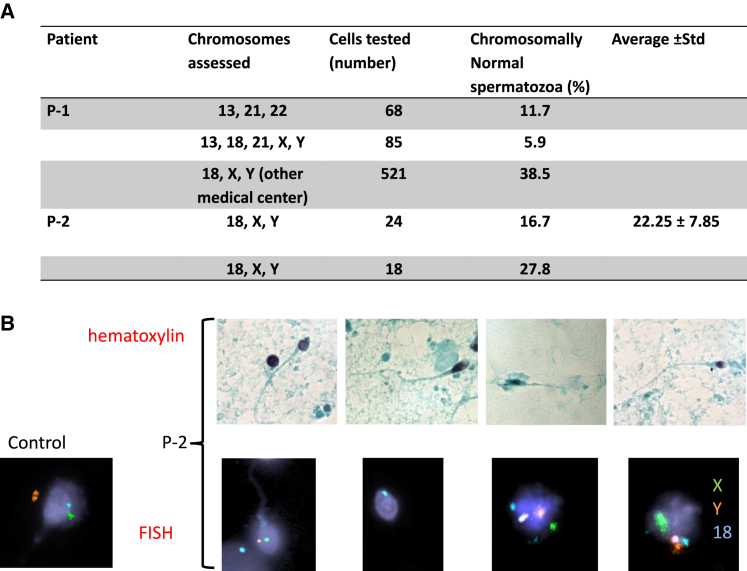
Patients' spermatozoa assessment (A) Summary of the fluorescent *in situ* hybridization (FISH) spermatozoa findings in P-1 and P-2. (B) Spermatozoa after H&E staining, ×1000; spermatozoa FISH with centromere probes for chromosomes X (green), Y (orange), and 18 (blue), ×400 in P-2 and control.

In accordance with the expression data, orthologous genes in both flies and mammals were reported to affect meiotic recombination rate, pairing of the homologous chromosomes, and/or crossover maturation.^38^^,^^39^^,^^40^^,^^41^^,^^42^^,^^43^ Furthermore, a previous study demonstrated dosage-dependent aneuploidy in *Rnf212* knockout mice.^44^ To more directly explore a possible role of RNF212B in meiotic recombination, we set out to examine the chromosomal ploidy status of sperm cells and embryos derived from our patients carrying the *RNF212B*^*C448T*^ variant. For this, we first performed FISH analyses on seven embryos derived from P-1 (five scored as 2PN and two as 1PN), using five autosomal probes (directed against chromosomes 13, 16, 18, 21, and 22). Significantly, analysis of 11 blastomere cells collectively taken from all the embryos exhibited various types of aneuploidy, including trisomies, monosomies, and nullisomies of autosomal chromosomes (Table S4). Similar analyses of sperm cells from this patient (68–521 sperm cells), using probes for three autosomal chromosomes (directed against chromosomes 13, 21, and 22) or probes for five sex and autosomal chromosomes (directed against chromosomes X, Y, 13, 18, and 21), accordingly revealed a very low percentage of sperm cells with a normal number of chromosome ploidy (11.7% and 5.9%, respectively), compared with 92%–98.2% of sperm cells with normal ploidy in patients attending our clinic, and the reported 98.68%–99.03% of sperm cells with normal ploidy in normozoospermic fertile men (Figure 3A and García-Mengual et al.^45^). Finally, 38% of all P-1 aneuploid sperm cells (aneuploidy tested for chromosomes 13, 18, 21, X, Y) contained both sex chromosomes, a rate that is much higher than the 5.2% rate in our unit, and 1.12% rate in García-Mengual et al.,^45^ indicating severe nondisjunction of the sex chromosomes.

Similar results were also obtained with sperm cells from P-2, using probes for the sex and autosomal chromosomes (directed against chromosomes X, Y, and 18). Since the sperm concentration of P-2 was very low, only about 20 sperm cells were analyzed in each experiment. Of these, only an average of 22% of the sperm cells had a normal number of chromosome ploidy (Figures 3A and 3B). Furthermore, aneuploidy of the sex chromosomes was observed in more than half of the analyzed sperm cells. Altogether, we conclude that aneuploidy is a major manifestation of spermatogenesis impairment in both brothers, usually occurring because of increased aberrations in meiotic recombination and chromosome synapsis, consequently leading to nondisjunction of the homologous chromosomes.

### The *Zip3/RNF212* gene family

The idea that mutations in human *RNF212B* and mouse *Rnf212* are both associated with aneuploidy raised questions about the molecular relationship between the two paralogs. We used phylogenetic analyses to examine the molecular evolution of the *RNF212* gene family. Whereas the presence of the two paralogs could be clearly detected in jawed vertebrates, including reptiles and birds, earlier diverging chordates (e.g., lampreys, lancelets, and tunicates) only have a single *RNF212*-like gene (Figure S4A and Table S5). The *RNF212* gene family is also present in other organisms, including multicellular animals, choanoflagellates (unicellular eukaryotes that are very close living relatives to animals), diverse fungi (including a single meiosis-specific gene, *CST9/ZIP3*, in the budding yeast *Saccharomyces cerevisiae*, involved in synaptonemal complex formation^46^^,^^47^), and even in some protists^48^ (Table S5).

This gene family encodes for proteins that are typically 200–400 amino acids in length, containing a common conserved region of about 120 amino acids in the N-terminal halves of the proteins, corresponding to an RING-HC Zn-finger domain^49^ (Figure S4B). Besides the gene duplication in jawed vertebrates, which occurred before the split of cartilaginous and bony fish, analysis of the relations between RNF212-like protein sequences suggests additional apparent independent gene duplications in diverse animal groups. For example, the presence of multiple paralogs in the nematodes^50^ and the previously noted emergence of *nenya* and *narya RNF212*-like genes from their *vilya* paralog in fruit flies.^51^ Furthermore, our analyses identified likely duplications of *RNF212*-like *vilya* and *nenya* genes in other flies and mosquitos, as well as duplications of *RNF212*-like genes in other arthropods (Figure S5), sponges, segmented-worms, and Xenacoelomorpha (Figure S6). Because of the independent duplications, RNF212-like proteins from different phyla do not significantly cluster with each other (Figure S6). Nevertheless, we found that RNF212B proteins in vertebrates lack a 17-amino acids motif present in the C-terminal halves of the vertebrate RNF212 proteins and in the single RNF212-like proteins of simple chordates (lampreys, lancelets, tunicates) and other diverse invertebrates, revealing a unique sequence signature that distinguishes RNF212-like proteins from RNF212B-like proteins (Figure S7, Table S5).

### The *Drosophila ZIP3/RNF212*-related gene family is involved in male and female fertility

**Figure 4:**
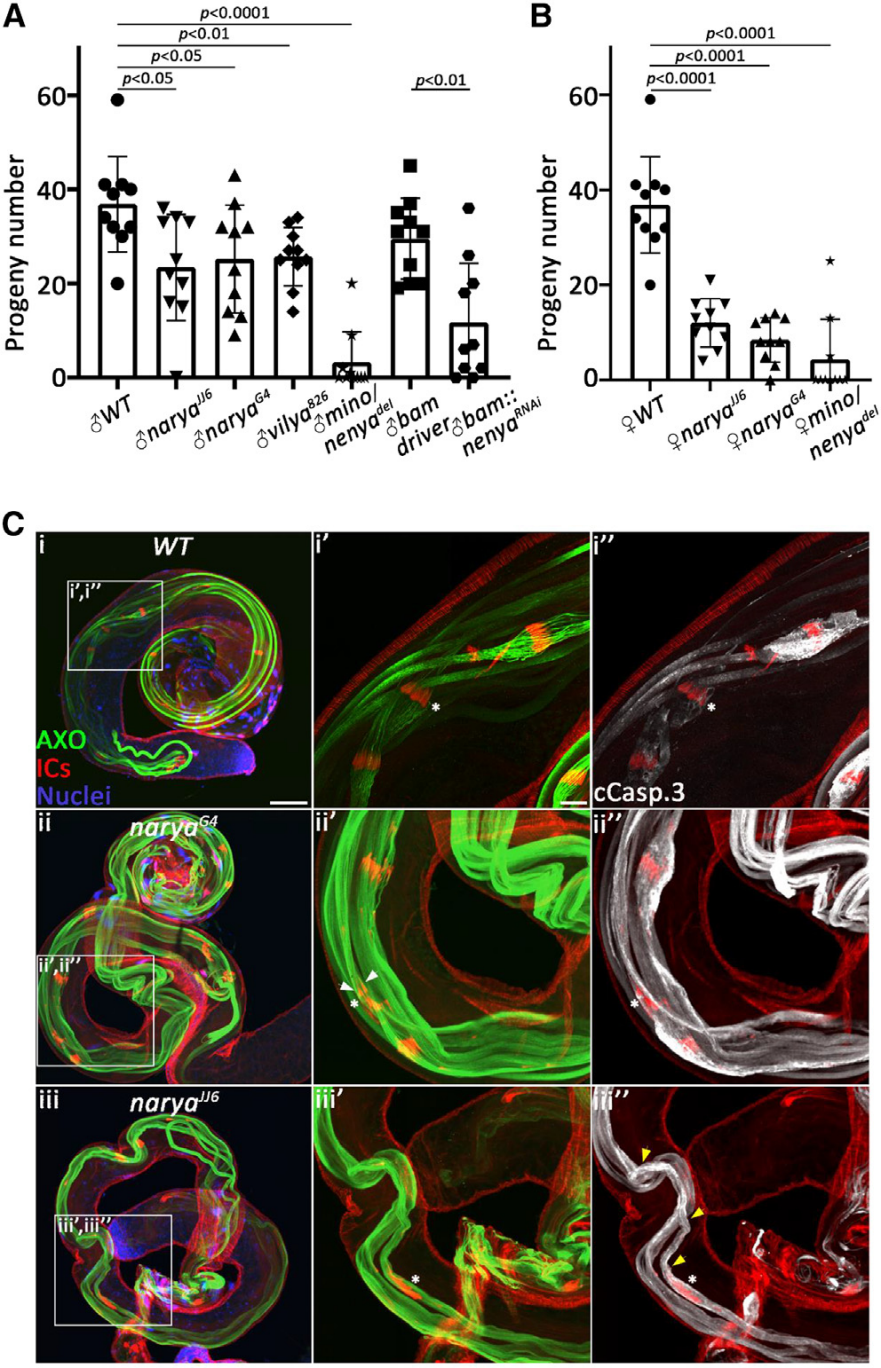
The *Drosophila* Zip3/RNF212 orthologs affect both male and female fertility (A) Male and (B) female fertility tests were performed for flies of the indicated genotypes. P values of the fertility differences compared with wild-type (WT) are indicated above the bars. N = 10 replicates/group. Data represent the mean ± SEM. Two-tailed Student’s t test. *mino/nenya*^*del*^, a complete deletion of the *nenya* gene that presumably also affects the *minotaur* gene. (C) Analysis of post-meiotic spermatid differentiation reveals that *narya* mutant testes display mild-moderate defects during the spermatid individualization process. Dissected WT (i) and mutant (ii, iii) testes of the indicated genotypes were stained with the anti-polyglycylated tubulin antibody to visualize the axoneme in individualizing spermatids (AXO; green), phalloidin to label the actin cones of the individualization complex (IC; red), DAPI to mark the nuclei (blue), and the anti-cleaved caspase-3 antibody to visualize the cytoplasmic contents of the individualizing spermatids (white; this channel is only shown in i", ii", iii"). To better visualize the specific individualization defects, selected areas in each testis, confined by white squares, were magnified and presented separately with letters corresponding to the letters within the white squares. (i', i") In the WT testis, the 64 actin-based cones that compose each IC translocate synchronously (a white asterisk in i'), expelling the spermatids' cytoplasmic contents into a cystic bulge (CB), which gradually grows in volume (a white asterisk in i"). (ii', ii", iii', iii") In contrast, the ICs in the *narya* mutants occasionally display severe asynchronization of the actin cones (a white asterisk in ii'), manifested by scattered actin cones with less than 64 of them in a CB (a white asterisk in iii'), and highly reduced volume of the CBs (white asterisks in ii", iii") due to failure of the ICs to properly extrude the spermatids' cytoplasmic contents. The retained cytoplasmic contents in the mutant spermatids are readily visualized with the anti-cleaved caspase-3 staining at regions rostral to the IC (yellow arrowheads in iii"). Occasionally, the asynchronization of the actin cones in the mutants is also manifested by actin cones pointing to the opposite direction (a white arrowhead in ii'). Scale bars in (i–iii), 30 μm; (i'–iii', i"–iii"), 10 μm.

The process of chromosome synapsis and genetic recombination during meiosis involves several crucial steps, including the pairing of the homologous chromosomes, crossover recombination, and segregation of the homologous chromosomes to different poles of the cell. To further refine the role of the *ZIP3/RNF212*-related gene family in this process, we turned to the fruit fly *Drosophila melanogaster*, in which, although the pairing of the homologous chromosomes is critical for proper chromosomal segregation in both sexes,^11^^,^^52^^,^^53^ meiotic crossover and recombination only occur during female meiosis and are completely absent in male meiosis (a phenomenon called achiasmy).^9^^,^^10^^,^^54^ Recent studies identified three *Zip3/RNF212*-related genes in *Drosophila*, called *vilya*, *narya*, and *nenya*.^51^^,^^55^ All three paralogs were reported to physically interact and function in the initiation of meiotic recombination in the pro-oocytes, promoting the formation of DNA double-strand breaks (DSBs) and the subsequent processing of selected DSBs into crossovers.^51^^,^^55^ However, the role of this gene family in *Drosophila* male fertility has not been reported. We therefore first performed fertility tests with both male and female flies homozygous for previously characterized mutant alleles of the three genes. Whereas females homozygous for a null allele of *vilya* (*vilya*^*826*^) reportedly displayed a decrease of 55% in the number of produced offspring,^55^ males carrying this allele exhibited less but still significant 30% reduction in fertility (Figure 4A). Likewise, flies homozygous for a null allele of *narya* (*narya*^*JJ6*^) or a *narya* RING mutant allele (*narya*^*G4*^),^51^ exhibited a 70%–80% reduction in female fertility and 30%–35% reduction in male fertility (Figures 4A and 4B). Interestingly, whereas a previous report demonstrated that *narya* and *nenya* are functionally redundant with respect to the formation of meiotic DNA DSBs in the pre-oocyte, and that knockout and knockdown of *nenya* alone had no effect on proper chromosome segregation during female meiosis,^51^ knockdown of *nenya* (using the same RNAi line) caused a significant 60% reduction in male fertility, and indeed had no effect on female fertility, suggesting that *nenya* is the predominant *RNF212*-like paralog in the male germ cells (Figures 4A and 4B). Of note, in contrast to the *nenya* knockdown results, flies homozygous for a reported null allele of *nenya* (*nenya*^*del*^; containing a deletion of the entire gene^51^) displayed almost complete female and male sterility (Figures 4A and 4B). We attribute this discrepancy to the fact that the *nenya* gene is located in the first intron of yet another gene, *minotaur* (*mino*),^51^ of which mutant alleles were previously reported to cause both female and male sterility.^56^

Accumulated data from multiple *Drosophila* mutant analyses indicate that spermatid differentiation can sometimes proceed even in the absence of landmark meiotic cytological events, such as chromosome segregation and cytokinesis, and sometimes also in mutants that skip meiosis altogether, and that the abnormalities were displayed only during later stages of spermatogenesis.^57^ Given that inactivation of each of the three *RNF212*-like paralogs affected *Drosophila* male fertility, but neither caused complete sterility, we reasoned that the mutant spermatids might also exhibit cytological defects. To examine this, testes from *narya*^*G4*^ and *narya*^*JJ6*^ recessive mutant flies were dissected and stained to visualize the post-meiotic differentiating spermatids. In accordance with the mild fertility reduction in these mutants, the testes were of regular size, containing grossly normal numbers of cysts of terminally differentiating spermatids, as detected by the presence of elongated spermatids positive for a late differentiation marker of the axoneme (AXO), which normally appears at the onset of spermatid terminal differentiation, a process also called spermatid individualization^58^^,^^59^ (Figures 4C and 4I, ii, iii). Spermatid individualization is driven by an actin-based individualization complex (IC), composed of 64 actin cones, one for each spermatid in a cyst, which move synchronously from the nuclei toward the caudal end of the cyst, investing each spermatid in its own plasma membrane and simultaneously extruding most of the syncytial cytoplasm into a membrane-enclosed structure called cystic bulge (CB), which eventually is pinched off the spermatids as waste bag.^60^ Closer examination of the individualizing spermatids, using phalloidin to stain the actin filaments of the ICs,^61^ revealed that in every mutant testis, but not in wild-type testis, at least one cyst of individualizing spermatids displayed an IC with severely asynchronous actin cones (Figures 4C and 4I', ii', iii'). Furthermore, staining these testes with the anti-cleaved caspase-3 antibody (cCasp.3) to detect the spermatids' cytoplasm, revealed CBs with highly reduced volume, as well as trails of cytoplasm in the post-individualized portion of the spermatids, indicating a failure of the spermatids to properly remove their cytoplasmic contents (Figure 4C, i", ii", iii"). Collectively, these findings suggest that the *Zip3/RNF212*-related genes in *Drosophila* may either be involved during the homologous chromosome pairing step and that the cytological abnormalities are manifested only during the post-meiotic stages, or that they may exert other function/s unrelated to meiosis during later spermatogenesis stages.

## Discussion

In this study, we investigated a case of familial OAT with repeated MAR failures and successfully uncovered a nonsense PV in the uncharacterized gene *RNF212B*. Although we cannot completely rule out some deleterious effects of the additional candidate variant in *ERCC4* on male gametogenesis, we believe that it is unlikely to play a significant role in the phenotypes observed in this study. First, the candidate variant in *ERCC4* is expressed in the testis and almost all other somatic tissues. Thus, one may expect that a deleterious variant in *ERCC4* may cause diseases reported in patients with ERCC4 malfunction (i.e., Xeroderma Pigmentosum, Fanconi Anemia Complementation Group F, and Group Q). However, none of these diseases have been associated with our patients. Second, RFLP screening of the *ERCC4* variant found PS-1 to be negative for this variant, even though PS-1 displays a spermatogenic phenotype reminiscent of the affected brothers, indicating that loss of RNF212B is sufficient to cause spermatogenic failure.

We further associated the loss of RNF212B function with impaired spermatogenesis and a high rate of aneuploidy in the affected sperm and the derived embryos, accounting for this severe familial case of male infertility. *RNF212B* is almost exclusively expressed in the human testis, and both *RNF212B* and its paralog, *RNF212*, share relatively high sequence similarity, mainly in the N′ terminal region, where they harbor a highly similar RING-HC domain. In accordance, both genes were previously associated with recombination rates in cattle, deer, and sheep.^38^^,^^39^^,^^40^ Furthermore, a previous study suggested that RNF212 controls chromosome pairing and crossover during meiosis, and that genetic variants in *RNF212* might be a risk factor for aneuploidy conditions.^44^ Our comparative genomics shows that the *RNF212*/*RNF212B* gene duplication in vertebrates occurred at the emergence of jawed vertebrates, as shown in the number of these genes in different species and the topology of the dendrogram calculated from their sequences. RNF212-like proteins are present in diverse eukaryotes, from protists to animals, with evidence that an early diverging ortholog ancestral gene is involved in recombination.^39^ A common sequence motif in *RNF212* of jawed vertebrates and in some earlier diverging members, which is missing in *RNF212B*, may suggest that these two genes also have some non-overlapping functions.

Our study provides compelling evidence for the involvement of RNF212B in human male meiosis, in sperm cell ploidy, and perhaps also in post-meiotic stages. First, scRNA-seq data analysis revealed *RNF212B* expression in spermatogonia, spermatocytes, and round spermatid cells, from the onset of meiosis I in differentiated spermatogonia, peaking during pre-leptotene, through zygotene to pachytene stages. Interestingly, unlike *RNF212, RNF212B* also retains low but significant expression levels in early and mid-round spermatids. Our findings demonstrate a significant overlap between the expression patterns of *RNF212* and *RNF212B* during the recombination-associated processes of meiosis prophase I, but not during earlier or post-meiotic stages. Nevertheless, although we cannot rule out the possibility of some post-meiotic impairments, we attribute the etiology for infertility in P-1 and P-2 mainly to the severe genome instability detected in sperm and embryos derived from these patients, which is consistent with a role of this family of proteins in the homologous recombination-related processes during meiosis I.^42^^,^^44^ Likewise, the *Drosophila Zip3/RNF212*-related genes may also function either during the pairing of the homologous chromosomes or at post-meiotic stages. Since an early role in homolog pairing or a late post-meiotic role has never been demonstrated for this family of proteins, identifying their exact role in *Drosophila* spermatogenesis shall shed light on a new or more refined function of these proteins during the homologous recombination stage, which could be then tested in higher model organisms. Given that E3 Small Ubiquitin-like Modifier (SUMO) ligase activity has been demonstrated for at least some members of the RNF212 protein family,^41^^,^^62^^,^^63^ and that the histone-to-protamine transition process in *Drosophila* has been associated with high expression levels of SUMO proteins,^64^^,^^65^ it would be intriguing to test the hypothesis that these proteins may have an additional role in the histone-to-protamine transition stage. Along these lines, it has been reported that DNA DSBs might facilitate chromatin unwinding as a prelude to protamine deposition,^64^^,^^66^^,^^67^ and accordingly, a role of RNF212-like proteins in the formation of DNA DSBs and crossover maturation in meiosis I of the *Drosophila* pre-oocyte was also demonstrated.^51^^,^^55^ Alternatively, experiments directed at assessing the ploidy of the *Drosophila* and/or mice spermatids would help determine possible earlier effects during pairing of the homologs.

Chromosome instability is well documented in severe cases of male infertility,^68^ as well as in other manifestations of decreased general health and increased morbidity.^69^ Idiopathic OAT caused by genome instability is believed to stem from several genetic and epigenetic factors, including PVs.^70^ Using FISH to assess aneuploidy in a large group of couples with a history of three or more IVF failures and/or miscarriages, but with normal somatic karyotypes, Magli et al. demonstrated that poor prognosis of term pregnancy, especially in cases with severe male factor, is usually associated with high proportions of spermatozoa that display chromosome abnormality.^71^ However, causative variants that may be responsible for these conditions have thus far not been associated with known PVs in meiosis-related genes, such as genes known to be involved in meiotic checkpoint and proper chromosomal pairing and segregation (e.g., *SYCP3*, *M1AP*, *TEX11*, *MEIOB,* and *GCNA*^15^^,^^72^^,^^73^^,^^74^^,^^75^), since they all lead to meiotic arrest and complete absence of sperm cells in the ejaculate.^13^^,^^14^^,^^31^^,^^73^^,^^76^ In contrast, the *RNF212B*^*C448T*^ variant is, to the best of our knowledge, the first mutation in an essential human meiotic recombination gene that still allows the production of mature sperm cells with extensive aneuploidy.

We found that an additional patient (PS-1) with multiple IVF failures was also homozygous for the same PV in P-1 and P-2. Unlike P-1 and P-2 (Turkish Jews), PS-1 is of Ashkenazi descent. A recent genome-wide study of samples from ancient Ashkenazi Jews indicated the highest genomic overlap with Turkish Jews (Sephardi Jews).^77^ This presumably indicates a common genetic pool between Sepharadi and Ashkenazi Jews, as previously suggested.^78^ The CNV analysis identified different haplotype backgrounds for P-1/P-2 and PS-1. A one-order of magnitude elevation in the population allele frequency of the *RNF212B*^*C448T*^ variant was found within the Ashkenazi Jewish population (N > 4,000 samples of Ashkenazi Jews) compared with all other populations. Altogether, a possible explanation is that the *RNF212B*^*C448T*^ variant resulted from an early founder event before the spread of Ashkenazi and Sephardi Jews all over Europe. However, further research is needed to examine this possibility. In Israel, couples can undergo an unlimited number of IVF cycles, which may last for decades. PS-1 was detected after screening only a small group of 13 patients with a reported history of repeated IVF-ICSI failures. This suggests that the *RNF212B*^*C448T*^ variant may be relatively frequent among European descendant Jews, and perhaps even more frequent in Jewish couples with repeated IVF-ICSI failures. Testing such infertile men can save years of frustrations due to futile multiple IVF cycles, and the couples may be directed to explore other options. This is particularly important for PVs in genes such as *RNF212B*, as even if some moderate PVs in these genes are tolerated during embryonic development, they could lead to significant fetal malformations due to genomic aberrations.

In current clinical practice, fertility evaluation of the male partner is measured by assessing the number of spermatozoa in the ejaculate and investigating sperm motility and morphology. Today, MAR treatments are of great help in overcoming male infertility, even in cases when only a few spermatozoa are detected. However, some couples face repeated MAR treatment failures, calling into question the usefulness of current semen analysis as an indication of appropriate insemination treatment for couples with unexplained infertility.^79^^,^^80^ Indeed, semen analysis rarely predicts the functionality or fertilization capacity of the male gamete, as no correlation has been observed between any of the morphological assessments of spermatozoa and fertilization rate, embryo score, or pregnancy rate after either IVF or ICSI.^81^ This is particularly evident in cases of unexplained infertility where both the male and female partners exhibit normal results in all conventional tests. Exploring genetic causes of male infertility and using additional tests to gain more insight into individual’s reproductive capacity is, therefore, of great clinical interest. Our study contributes to the understanding and diagnosis of couples with male factor and recurrent IVF failures and demonstrates the role of previously uncharacterized *RNF212B* in human meiosis and male fertility.

## Ethics approval and consent to participate

All study participants consented to undergo genetic evaluations and signed written informed consent. The local institutional review board committee approved the study in accordance with the Helsinki Declaration of 1975.

## Data Availability

The complete exome sequences of the patients that were produced and analyzed are available from Tel Aviv Sourasky Medical Center, but restrictions apply to the availability of these data, which were used under license for the current study, and so are not publicly available. Data are, however, available from the authors upon reasonable request and with permission of Tel Aviv Sourasky Medical Center. All other datasets are included in this published article and its supplementary information files or publically available. BWA:GitHub: https://github.com/lh3/bwa. Picard Tools:GitHub: https://broadinstitute.github.io/picard/. GATK software:GitHub: https://github.com/broadinstitute/gatk/releases. scRNA-seq database:Mendeley Data: https://data.mendeley.com/datasets/kxd5f8vpt4/1. MORPHEUS software: https://software.broadinstitute.org/Morpheus. 10X Genomics Loupe cell browser: https://support.10xgenomics.com/single-cell-gene-expression.

## Web resources

GeneCards: https://www.genecards.org/.

Webcutter program: http://www.firstmarket.com/cutter/cut2.html.

Online Mendelian Inheritance in Man (OMIM): https://www.ncbi.nlm.nih.gov/omim/.

## Supplemental information

Document S1. Figures S1–S7 and Tables S1–S4

Table S5. RNF212-like.sequence_data

Document S1. Article plus supplemental information
