# Supplementary material for: A pathogenic variant in the uncharacterized RNF212B gene results in severe aneuploidy male infertility and repeated IVF failure
Source: HGG Adv. 2023 Mar 31;4(3):100189. doi: 10.1016/j.xhgg.2023.100189 (PMC10133878; doi:10.1016/j.xhgg.2023.100189)
Supplement: Document S1. Figures S1–S7 and Tables S1–S4 [file mmc1.pdf]

HGGA, Volume *4*

Supplemental information

A pathogenic variant in the uncharacterized *RNF212B* gene results in severe aneuploidy male infertility and repeated IVF failure

Moran Gershoni, Tslil Braun, Ron Hauser, Shimi Barda, Ofer Lehavi, Mira Malcov, Tsvia Frumkin, Yael Kalma, Shmuel Pietrokovski, Eli Arama, and Sandra E. Kleiman

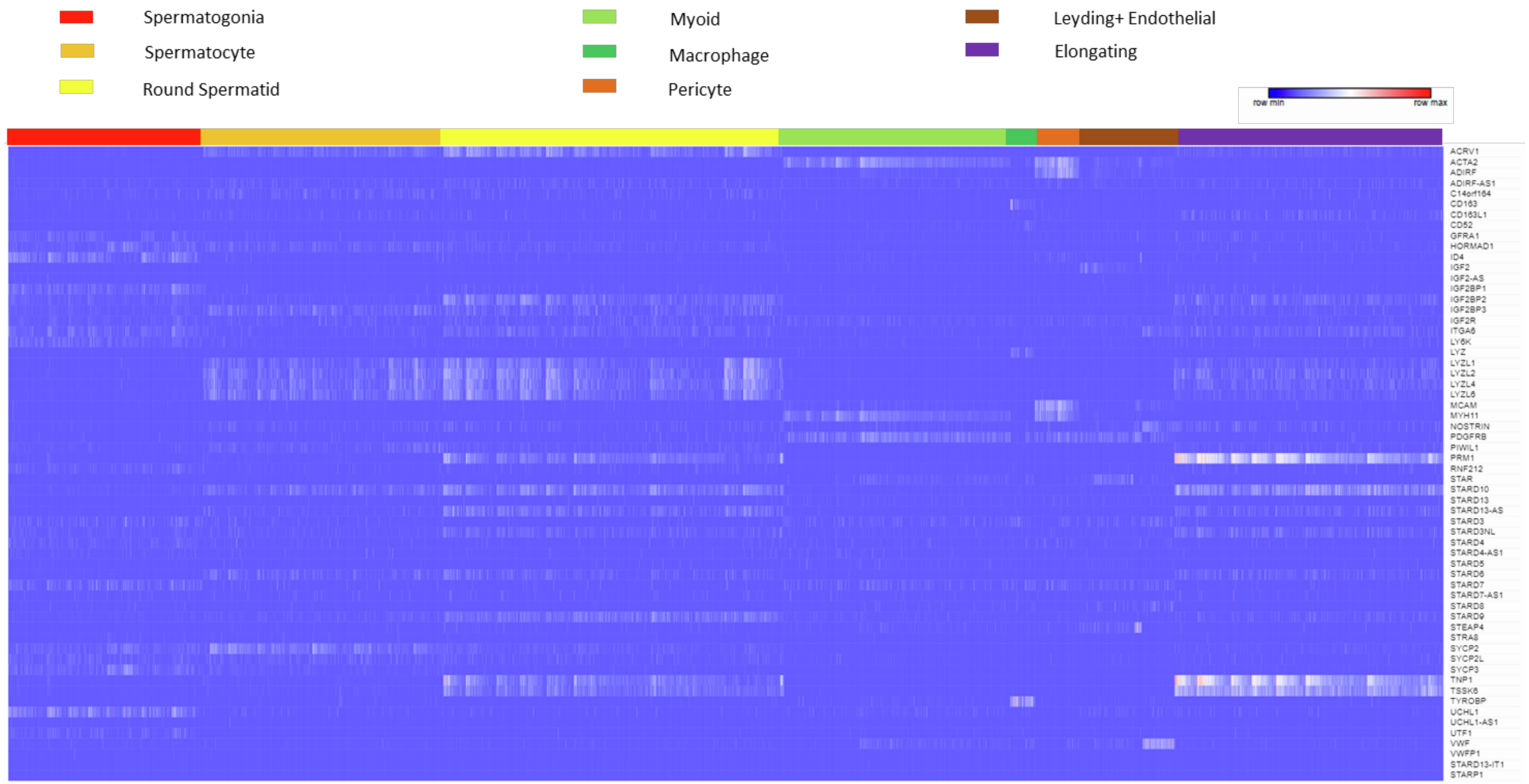

Figure S1. Hierarchical clustering of testis single-cell RNA-seq obtained from[1] using spermatogenic cell markers (X axis, as detailed in the Materials and Methods section).

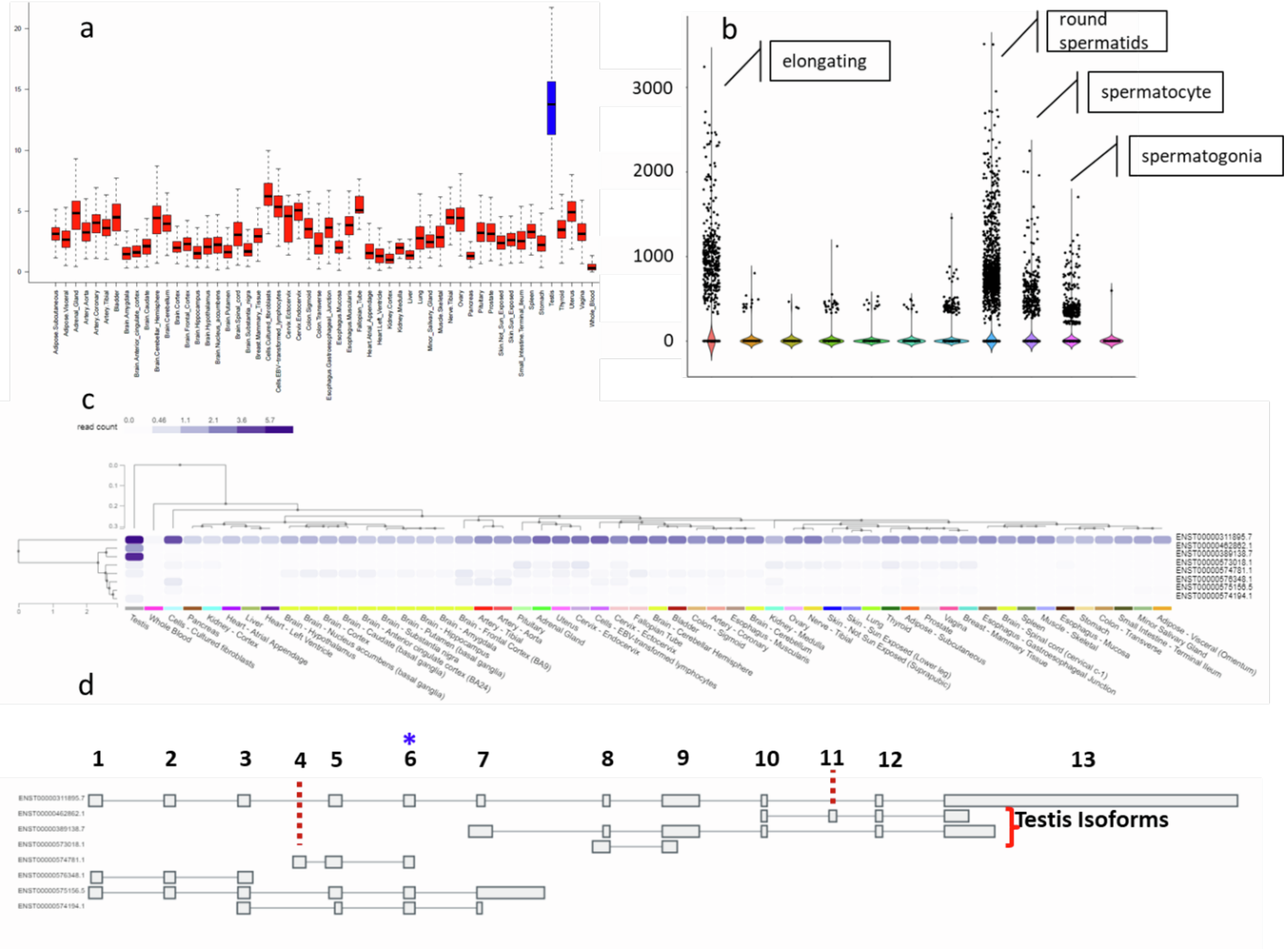

Figure S2. ERCC4 expression analysis. a) Expression pattern of ERCC4 across 53 human tissues obtained from GTEx[20], blue bar denotes testis tissue. b) Testis single-cell RNA-seq analysis of ERCC4 shows increased expression levels in round spermatids and elongating cells. c) Expression of ERCC4 transcript isoforms shows two ERCC4 testis-specific transcripts. d) schematic structure of ERCC4 as inferred from the GTEx data shows that

ERCC4 testis-specific isoforms do not harbor the exon with the candidate variant. The Blue asterisk denotes the variant location. Dashed lines point to the exon number.

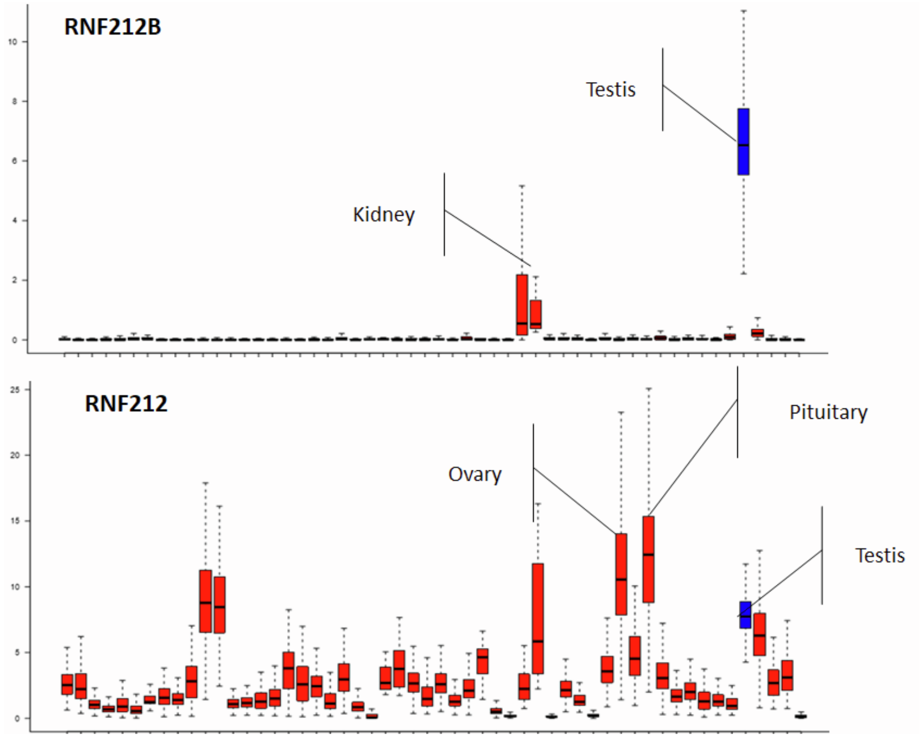

Figure S3. RNF212B bulk RNA-seq expression analysis. Expression of RNF212B and RNF212 in Transcript per million (TPM) values (Y-axis) across 53 human tissues (X-axis). Blue bars denote testis tissue. RNF212B shows almost testis-exclusive expression (with some expression in two kidney tissues), contrary to RNF212, which shows high expression in Ovary and several somatic tissues.

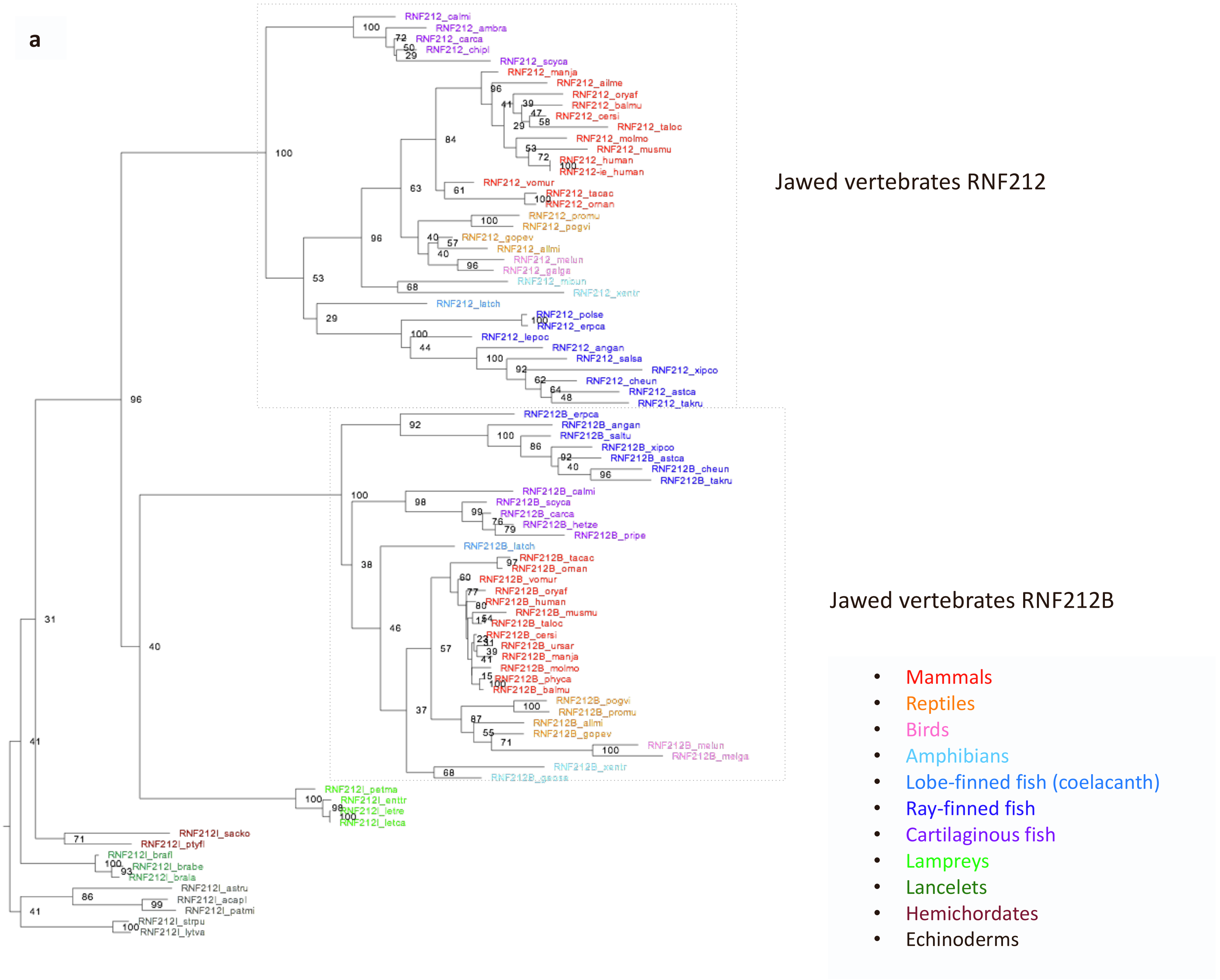

a
Jawed vertebrates RNF212
Jawed vertebrates RNF212B
RNF212_calmi
RNF212_ambra
RNF212_carca
RNF212_chipl
RNF212_scyca
RNF212_manja
RNF212_ailme
RNF212_oryaf
RNF212_balmu
RNF212_cersi
RNF212_taloc
RNF212_molmo
RNF212_musmu
RNF212_human
RNF212-ie_human
RNF212_vomur
RNF212_tacac
RNF212_ornan
RNF212_promu
RNF212_pogvi
RNF212_gopev
RNF212_allmi
RNF212_melun
RNF212_galga
RNF212_micun
RNF212_xentr
RNF212_latch
RNF212_polse
RNF212_erpca
RNF212_lepoc
RNF212_angan
RNF212_salsa
RNF212_xipco
RNF212_cheun
RNF212_astca
RNF212_takru
RNF212B_erpca
RNF212B_angan
RNF212B_saltu
RNF212B_xipco
RNF212B_astca
RNF212B_cheun
RNF212B_takru
RNF212B_calmi
RNF212B_scyca
RNF212B_carca
RNF212B_hetze
RNF212B_pripe
RNF212B_latch
RNF212B_tacac
RNF212B_ornan
RNF212B_vomur
RNF212B_oryaf
RNF212B_human
RNF212B_musmu
RNF212B_taloc
RNF212B_cersi
RNF212B_ursar
RNF212B_manja
RNF212B_molmo
RNF212B_phyca
RNF212B_balmu
RNF212B_pogvi
RNF212B_promu
RNF212B_allmi
RNF212B_gopev
RNF212B_melun
RNF212B_melga
RNF212B_xentr
RNF212B_geosa
RNF212l_petma
RNF212l_entr
RNF212l_letre
RNF212l_letca
RNF212l_sacko
RNF212l_ptyfl
RNF212l_brafl
RNF212l_brabe
RNF212l_brala
RNF212l_astru
RNF212l_acapl
RNF212l_patmi
RNF212l_strpu
RNF212l_lytva
Mammals
Reptiles
Birds
Amphibians
Lobe-finned fish (coelacanth)
Ray-finned fish
Cartilaginous fish
Lampreys
Lancelets
Hemichordates
Echinoderms

b

```
RNF212B   2 D--W-FHCNQCFRK-----DGAHFFVTSCGHIFCKKCV---TLE------------KCAV  38
RNF212    3 N--W-VFCNRCFQPPH-RT--SCFSLTNCGHVYCDACLGKGKKN------------ECLI  44
Vilya    15 SKLW-IHCNSCCALF-CDKKHTFFLLA-CHHVFCERCVKV----SAGRTPSDAPIFECST  67
Nenya     2 ---FRIHCNKCFRRR-NVEPTLIFHMTQCQHVLCASCLSES---S-TDK-------KCPL  46
Narya     2 ---FRVHCNKCFRHR-KTDPAVPFHLTQCRHVICGPCLGQS---S-LEK-------NCPL  46

RNF212B  39 CGTACKHLALSDNLKPQEKMFFK-SPVETAL-QYFS-H---ISQVWSFQK-KQTDLLIAF  91
RNF212   45 CKAPCRTVLLSKHTDADIQAFFM-SIDSLCK-KYSR-E---TSQILEFQE-KHRKRLLAF  97
Vilya    68 CRRSVRGRQLTNSMPNHFKQLFHPEP-----F---TIGNDF---VETFQRGNHR-HFDKY 115
Nenya    47 CKRDLRAIPIDKNLPPNVAQYFE-DP-----LRF-QQLYRK---ISKFQ-ADQRASDNLG  95
Narya    47 CGQVLKAIQINRDMPTSVANYFA-DP-----LRF-QQIYRK---ISKFQ-ADQRASDNLG  95

RNF212B  92 YKHRITKLETAMQEAQQALVSQ---------DK                           115
RNF212   98 YREKISRLEESLRKSVLQIEQL---------QS                           121
Vilya   116 KERKELEMDKLFKDIEVAKSVCQKRFLEAQ--                            145
Nenya    96 FYR---QMQEHEKNESRLKGFCKMEAQFNQ--                            122
Narya    96 FYR---QLQQLEQNKRQLEGFCKMEAQLNQ--                            122
```

Figure S4. RNF212-like proteins from vertebrates, hemichordates, and echinoderms. a) Dendrogram calculated from the RING zinc-finger sequence domain of representative proteins from species of various taxa, as denoted in the figure inset. Bootstrap values out of 100 are marked for each junction. The jawed vertebrate regions are marked and labeled. The Echinoderm cluster was used as an outgroup to root the tree. Note the congruence of the topology throughout the tree with established taxonomy of the different well-supported clusters. Sequence sources and descriptions are in Table S5. b) RING zinc-finger domains of Human RNF212 and RNF212B, and Drosophila melanogaster Vilya, Nenya, and Narya proteins. This domain is common in all RNF212-like protein family members.

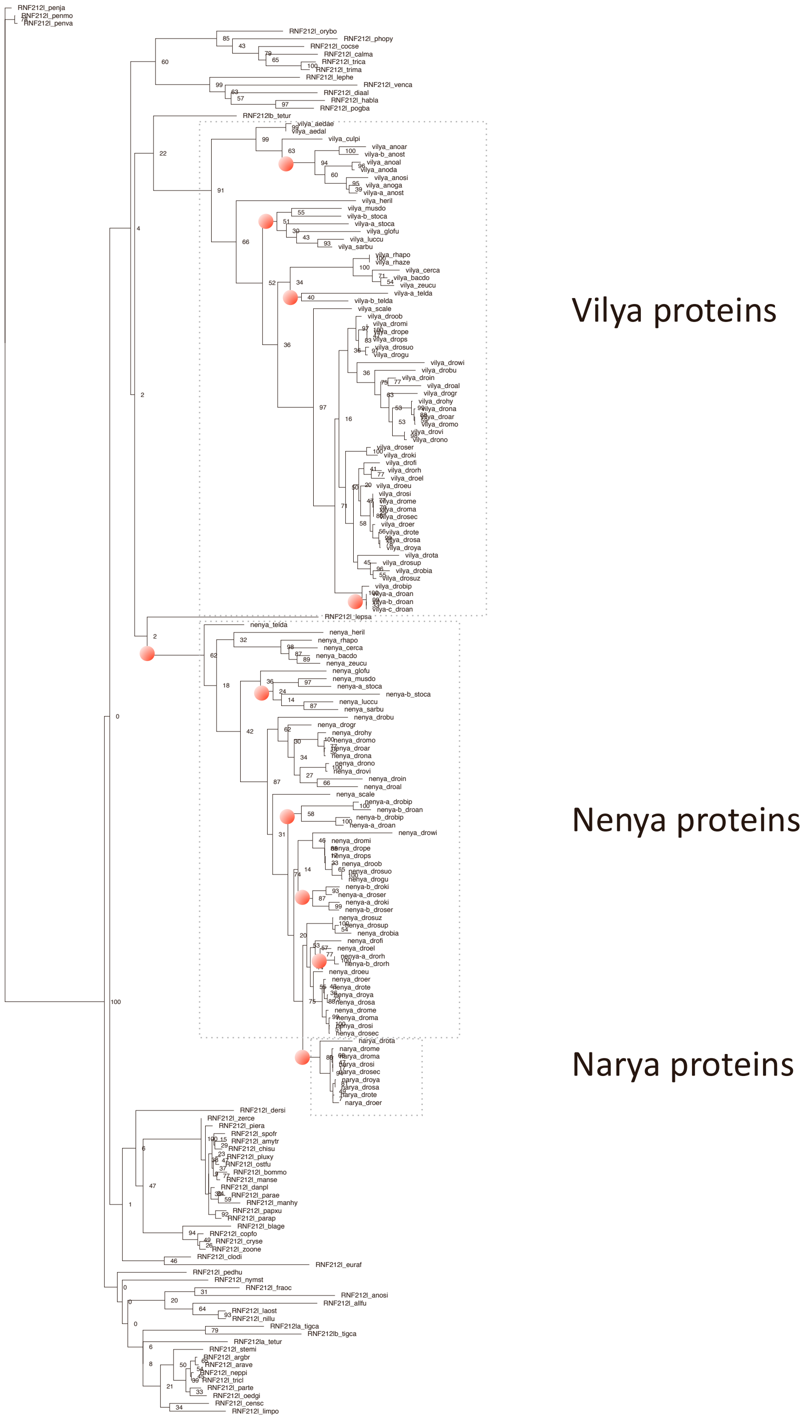
Vilya proteins
Nenya proteins
Narya proteins

Figure S5. Arthropods' RNF212-like proteins dendrogram. Regions of Vilya, Nenya, and Narya proteins are marked and labeled. Red spheres mark likely positions of gene duplications. Bootstrap values out of 100 are marked for each junction. Sequence sources and descriptions are in Supplementary Table 5.

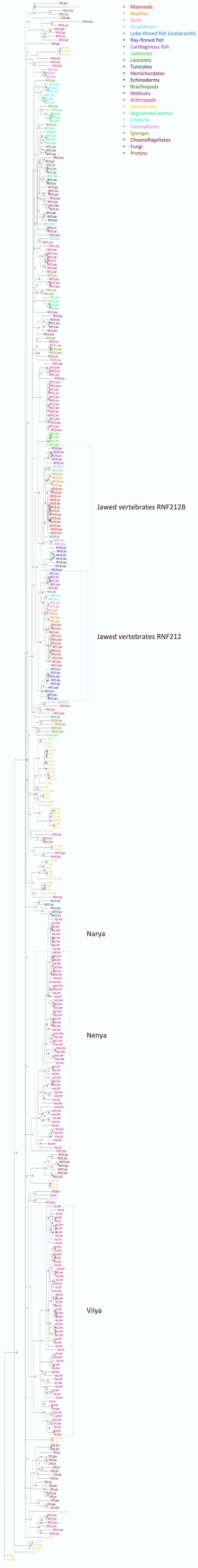
Mammals
Reptiles
Birds
Amphibians
Lobe-finned fish (coelacanth)
Ray-finned fish
Cartilaginous fish
Lampreys
Lancelets
Tunicates
Hemichordates
Echinoderms
Brachiopods
Mollusks
Arthropods
Nematodes
Segmented worms
Cnidaria
Ctenophores
Sponges
Choanoflagellates
Fungi
Protists
Jawed vertebrates RNF212B
Jawed vertebrates RNF212
Narya
Nenya
Vilya

Figure S6. Sequence relation between RNF212-like proteins (appendix) . Dendrogram calculated from the RING zinc-finger sequence domain (figure S4b of diverse proteins, colored by phylogenetic groups as shown in the inset). Bootstrap values out of 100 are marked for each junction. The jawed vertebrate regions, and arthropod Vilya, Nenya, and Narya-like proteins are marked and labeled. Sequence sources and descriptions are in Supplementary Table 5.

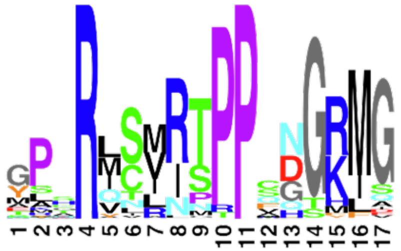

Human	176	GPARISMISPPQDGRMG
Lamprey	223	GSSRVSMRNPPAEGRMG
Lancelet	210	GPARITMRTPPIDGKMG
Tunicate	212	EKPRMSLRSPPVGGKIQ
Acorn worm	220	GPGRITMRTPPVDGKMG
Sea-urchin	217	GPARYCIRTPPSGGRMG
Spider	191	LPQRMCVITPPMNGKLG
Octopus	234	MSSRVSIRTPPSQGRID
Leech	233	GPSRLSMRTPPINGRIA
Brachiopod	196	MASRLSVRTPPVHGRLG
Coral	202	GPTRLSVRTPPCNGFMG
Hydra	255	SNGRISLRTPPVNGKLG
Sponge	356	APGRLSIRTPPINGKMG

Figure S7. Protein sequence motif present in RNF212 proteins of jawed vertebrates and other animals. Top, sequence logo of the motif. Bottom, motif sequences and protein start coordinates from representative species: Human (accession NP_001353848.1),

lamprey (*Petromyzon marinus*, XP_032830699.1), lancelet (*Branchiostoma floridae*, XP_035678696.1), tunicate (*Ciona intestinalis*, XP_002131044.1), acorn worm (*Saccoglossus kowalevskii*, XP_006816460.1), sea-urchin (*Strongylocentrotus purpuratus*, XP_030831903.1), spider (*Nephila pilipes*, GFS75922.1, octopus (*Octopus sinensis*, XP_029633755.1), leech (*Hirudo medicinalis*, MPNW01000810.1), brachiopod (*Lingula anatina*, XP_013394848.1), coral (*Pocillopora damicornis*, XP_027037663.1), hydra (*Hydra vulgaris*, XP_012561882.1), sponge (*Amphimedon queenslandica*, XP_019860695.1).

Supplementary Table 1. PCR primers, annealing temperature, product size, and fragments size after restriction treatment for detection of candidate mutations

GENE	Variant	Objective	Primers (5' -> 3')	PCR		Restriction ^		
				Annealing (^{o}c)	Product size (bp)	Enzyme	Normal allele (bp)	Mutant allele (bp)
RNF212B	Exon8:c.C448T:p.R150X	PCR, sequencing and RFLP	F- GTTGGAGAGCAATGGGTTTT<br>R- GGCCAATTTGTTGTACATGG	60	399	Taq I	208+235	399
		Rt-PCR	F- CTTCACCGGCTTCAACTCAT<br>R- GCAGGACTGGTAGCTGGAAA	64	cDNA:182<br>gDNA:1730			
ERCC4	exon6:c.A1062C:p.K354N	PCR and sequencing	F- GGAAGACAGGATGACAGCCA<br>R- ACACACTTTTCACATGGCCA	60	341			
		RFLP	mF- TTCCAGATGCCAAAATGAGTAA**G**A	60	170	MBO II	34+36+100	136+34
VCX3B	exon3:c.C385G:p.Q129E	PCR and sequencing	F- AAAGGGGCTGCGACAAAGAT<br>R- ATAGGGGAGTACTTGGCCGT	62	605+485			

^After restriction, PCR products were analyzed on agarose gel (3 %).

Both enzymes were from NEB (USA).

F, forward primer; R-reverse primer; mF mismatch forward primer; bp, base pairs.

Supplementary Table 2. CNVs analysis around RNF212B R150X PV

	**Location at chromosome 14**												
**Men tested**	21769717	22110223	22507947	22687399	22781471	23026660	23069623	23295947	**23731887**	23904379	24042993	24339720	24397229
									**RNF212B-mut**				
**P-1**	**363/363**	**270/270**	**286/286**	**313/313**	**426/426**	**327/327**	**275/275**	**225/225**		**266/266**	**251/251**	**323/323**	**233/233**
**P-2**	**363/363**	**270/270**	**286/286**	**313/313**	**426/426**	**327/327**	**275/275**	**225/225**		**266/266**	**251/251**	**323/323**	**233/233**
**Fertile brother**	**363**/366	**270**/270	**286**/292	**313**/313	**426**/428	**327**/333	269/**275**	**225**/229		**266**/266	**251**/251	**323**/323	**233**/243
**PS-1**	**363/366**	**270/276**	**280/284**	**313/326**	**428/428**	**327//327**	**275/275**	**225/225**		**266/278**	**251/251**	**323/323**	**241/249**

Supplementary Table 3. Likely functional candidate mutations in patients P-1 and P-2

**Chr**[1]	**Start**[2]	**Ref**[3]	**Alt**[4]	**GENE**[5]	**FUNC**[6]	**SIFT**[7]	**Mut. Taster**[8]	**gnomAD exome**[9]	**gnomAD genome**[10]	**Testis r-score**[11]
14	**23731887**	**C**	**T**	**RNF212B**	**stop**	**.**	**A**	**0.0002**	**.**	**0.99**
16	**14026102**	**A**	**C**	**ERCC4**	**NS**	**D**	**D**	**.**	**.**	**0.75**
**20**	47614885	G	A	ARFGEF2	NS	D	D	0.001	0.0003	0.21
**2**	1.49E+08	G	A	MBD5	NS	T	D	0.0023	0.0017	0.11
**2**	1.61E+08	C	T	ITGB6	NS	D	D	0.0081	0.0035	0.09
**20**	33511162	G	A	ACSS2	NS	T	D	0.0006	0.0002	0
**20**	44470575	G	A	ACOT8	NS	D	D	0.005	0.0044	0
**20**	40049450	T	A	CHD6	NS	D	N	0.0052	0.0029	-0.009
**1**	1.86E+08	G	A	HMCN1	NS	T	D	0.0009	0.0002	-0.02
**1**	1.86E+08	C	T	HMCN1	NS	D	D	0.0009	0.0001	-0.01
**6**	90121647	-	TCC	RRAGD	INS					-0.02
**20**	25060143	G	C	VSX1	NS	T	D	0.0034	0.0029	-0.02
**2**	1.61E+08	A	G	LY75	NS	D	N	3.25E-05	.	-0.04
**2**	1.67E+08	C	T	SCN7A	NS	D	N	4.08E-05	6.46E-05	-0.04
**X**	1.14E+08	G	A	LRCH2	NS	D	D	0.0003	0.0001	-0.06

1-2.Chromosomal position.

3. Reference allele (hg19).

4. Alternative allele.

5. HGNC gene symbol.

6. Transcriptional consequence. NS=non synonymous; INS=insertion; stop= stop-gain

7-8. functional impact prediction by SIFT[7] and Mutation tester[8] .D= deleterious; A= affect; T= tolerant; N= neutral

9-10. minor allele frequencies obtained from the gnomAD[45]

11. The Pearson correlation co-efficiency score for testis-specific expression (see material and method).

Supplementary Table 4. FISH findings of blastomeres after fertilization with P-1 sperm cells

Embryo number	Stage	Blastomeres tested	Fish findings
1	I	2	t21, m22
2	I	1	m13, m18, m22
3	I	2	m13, m22
4	I-II	2	m16, m18, m22, n13
5	I	1	t13
6	I arrest	2	m16, m18 ,n22
7	II-III arrest	1	haploid cell

A sampling of embryos was performed on day 3. The assessment was for chromosomes 13, 16, 18, 21 and 22

Stage I: <10% fragmentation and similar cell sizes. Stage II: 10-20% fragmentation, and cells were not all of the similar sizes.

t: trisomy

m: monosomy

n: nullisomy
